# A small molecule inhibitor of Nox2 and Nox4 improves contractile function after ischemia–reperfusion in the mouse heart

**DOI:** 10.1038/s41598-021-91575-8

**Published:** 2021-06-07

**Authors:** Ferenc L. M. Szekeres, Erik Walum, Per Wikström, Anders Arner

**Affiliations:** 1 Division of Genetic Physiology, Department of Physiology and Pharmacology, Karolinska Institutet, von Eulers Väg 8, 17177 Stockholm, Sweden; 2 Glucox Biotech AB, Frälsegårdsvägen 8, 179 97 Färentuna, Sweden; 3 Division of Biomedicine, Department of Health and Education, University of Skövde, Högskolevägen 1, 541 28 Skövde, Sweden; 4 Department of Clinical Sciences Lund, Thoracic Surgery, Lund University, c/o Igelösa Life Science AB Igelösa 373, 225 94 Lund, Sweden

**Keywords:** Cardiology, Diseases, Health care, Medical research

## Abstract

The NADPH oxidase enzymes Nox2 and 4, are important generators of Reactive oxygen species (ROS). These enzymes are abundantly expressed in cardiomyocytes and have been implicated in ischemia–reperfusion injury. Previous attempts with full inhibition of their activity using genetically modified animals have shown variable results, suggesting that a selective and graded inhibition could be a more relevant approach. We have, using chemical library screening, identified a new compound (GLX481304) which inhibits Nox 2 and 4 (with IC_50_ values of 1.25 µM) without general antioxidant effects or inhibitory effects on Nox 1. The compound inhibits ROS production in isolated mouse cardiomyocytes and improves cardiomyocyte contractility and contraction of whole retrogradely (Langendorff) perfused hearts after a global ischemia period. We conclude that a pharmacological and partial inhibition of ROS production by inhibition of Nox 2 and 4 is beneficial for recovery after ischemia reperfusion and might be a promising venue for treatment of ischemic injury to the heart.

## Introduction

The heart is an aerobic muscle—its contractile function is critically dependent on a continuous blood flow and an adequate supply of oxygen throughout life. These important processes are threatened in several common chronic diseases, associated with alterations in the coronary vessels, e.g., in atherosclerosis/ischemic heart disease which affects a large number of individuals and is one of the main causes of sudden death worldwide^1,2^. Although strong efforts are made to identify, prevent and treat contributing and confounding factors, like diabetes, hypertension and metabolic disorders, coronary atherosclerosis, conditions with cardiac ischemia/infarction and failure are still prevalent and constitute a severe burden in our society. In the acute situation, establishment of blood flow and oxygen supply to the heart is a primary goal of therapeutic interventions, but the combination of ischemia and reperfusion is a serious threat with generation of Reactive Oxygen Species (ROS) being a key pathological mechanism^3–7^. Although ROS can introduce cellular injuries, they still have beneficial effects in some organs, concentrations and time periods during an ischemia reperfusion challenge, which suggests that therapies targeting ROS should be specific, adequately dosed and introduced in a correct time period as pointed out by e.g.^8^.

Normal cellular processes use reactive oxygen (ROS) to maintain proper metabolism. Pathological overproduction by one or several of the specific sources of ROS is a common cause of toxic reactions and cell death. Several cellular processes can generate reactive oxygen species including the mitochondrial electron transport chain, xanthine oxidase, nitric oxide synthases, CP450, Cyclooxygenase, monoamine oxidase, NADPH oxidase family enzymes and also via succinate through reoxidation by succinate dehydrogenase (SDH) in the mitochondria^9–11^. NADPH oxidases (Nox) are a group of isoenzymes with the potential to produce large amount of ROS and may produce harmful levels of ROS when the cellular antioxidant capacity is exceeded. The Nox family enzymes are the only enzymes where production of reactive oxygen species is the primary function. To date seven members of this Nox family have been identified: Nox1, Nox2, Nox3, Nox4 Nox5, Duox1 and Doux2. Nox1, Nox2 and Nox3 have the most similarity among the isoforms, generate superoxide O_2_ and are activated by association with regulatory subunits^12–14^. Nox4 is widely expressed in the body and is constitutively activated and its cellular activity regulated through induced protein expression and^15,16^. Nox5 can be found mainly in spleen and testis and to lesser extent in other tissues of the body, e.g., tumor cells of different origin^17^. Hydrogen peroxide produced in excess by Nox4 contributes e.g., to glutamate mediated neurotoxicity and a number of medical conditions^18–22^. Duox 1 and 2 are regulated with Duoxa1 and 2 as well as calcium and they produce hydrogen and have originally been found in thyroid and now also in some other organs, but not in heart or brain^23^. Presently some of the Nox-isoforms (Nox1, Nox2 and Nox4) seem to be involved in various diseases, causative or contributing to the pathological process^24,25^.

In the heart, Nox2 and Nox4 are the main Nox isoforms expressed^6^. Nox4 has the most extensive tissue distribution and is located in endoplasmic reticulum (ER) and mitochondria and is induced by e.g., mechanical stress, hypoxia, ischemia, ER stress and mechanical stress^26–29^. Hypoxia inducible factor (HIF) is a main regulator at hypoxic conditions and regulates a vast number of genes and HIF is closely related to the Nox4/Nox2 activity^30^. The correlation of H_2_O_2_ with activation of neurofibromin 2 (NF2) and ischemia–reperfusion injury (I/R) injury indicates that this mechanism has a potential role in apoptosis of cardiomyocytes^31^. Although Nox activity thus seems to have an important role in the cardiac response to ischemia reperfusion challenges, the detailed function is not known. Knock down of Nox 2 using an antisense strategy attenuated hypoxia induced stress and apoptosis^32^. Using genetically modified animals where Nox 2 and 4 have been ablated, positive effects with reduced infarct size have been reported^6^, although effects were only seen in Nox 1 and 2 knockout animals in other studies^33,34^. The variable results from knockout animal studies suggest that full Nox inhibition might be a less successful approach and that the inhibition should be fine-tuned to have optimal effect in reducing ischemia reperfusion injury. In this study we have developed a new specific small molecule Nox2/Nox4 inhibitor (GLX481304), and show, using isolated cardiomyocytes and perfused whole hearts from mouse that the compound inhibits ROS production in the cardiac cells and improves contractility after an ischemia–reperfusion challenge.

A preliminary report of some of the results have been presented previously^35^.

## Materials and methods

### Animals and preparation

Female and male adult C57BL/6 mice were used (age 15–35 weeks, weight 20–36 g), randomly distributed into the experimental groups. Mice were maintained on a 12-h light/dark cycle and had free access to standard chow and water. Animals were euthanized with cervical dislocation according to approved procedures, the rib case opened, and a heparin/saline solution was injected in the aorta. Thereafter the heart was quickly excised, put into a beaker with cold (4 °C) Krebs–Ringer solution and transported to the experimental set-up. Cardiac perfusion was initiated within less than 7 min after euthanasia and the preparations were used for isolation of cardiomyocytes or evaluation of the contractile function in the intact heart.

### Isolation of cardiomyocytes and hypoxia/reoxygenation challenge

Cardiomyocytes were isolated as described previously^36^. The aorta was cannulated using a Langendorff system, and the heart was perfused with 37 °C pre-warmed perfusion buffer containing 2,3-butanedione monoxime (BDM) (10 mM) for 4 min at a flow of 3 ml/min to flush blood from the vasculature and relax the muscle. Digestive enzymes were then introduced in the BDM-containing perfusion buffer with 12.5 μM Ca^2+^ for 10 min at 3 ml/min. The heart was removed and the atria and the right ventricle were dissected away. The left ventricle was cut in pieces with scissors and further pulled apart with forceps into small pieces. Cells were dissociated using gentle pipetting with a 5 ml plastic pipette. Digestion was stopped by adding bovine calf serum. Large tissue pieces were then filtered away with a mesh, and Ca^2+^ was slowly reintroduced in perfusion buffer with bovine calf serum to a final concentration of 1 mM.

The hypoxia and reoxygenation challenge of cardiomyocytes was performed at 37 °C. The freshly isolated cell suspensions were exposed to 60 min of hypoxia in glucose free, Ca^2+^-containing (1 mM) perfusion buffer superfused with N_2_/CO_2_ (95%/5%). The glucose free condition was introduced to make hypoxic injuries more stable and mimic ischemia in vivo^37^. Non-hypoxic controls were held in the same solution with glucose exposed to air for same amount of time. After the 60 min period in hypoxia or normoxia, both groups were exposed to solution equilibrated with air and held at 37 °C for another 120 min in glucose containing, Ca^2+^-containing (1 mM) perfusion buffer during gentle shaking every 15 min. To address the question whether the GLX481304 Nox inhibitor influenced the properties of the cardiac cells following a challenge with hypoxia-reoxygenation, cells were treated during the hypoxia reoxygenation periods as described above in the presence of 1.25 µM GLX481304 or solvent control (0.1 mM DMSO). The loading of the Reactive Oxygen Species (H_2_DCFDA) and Ca^2+^ (FLUO-4) sensors were performed immediately after the reoxygenation period and the responses recorded as described below.

### Reactive oxygen species generation in stimulated cardiomyocytes

The cells were loaded for 30 min in perfusion buffer (1.0 mM Ca^2+^) at room temperature with the free reactive oxygen species indicator (DCF) Carboxy-H_2_DCFDA (5-(and-6)-carboxy-2',7'-dichlorodihydrofluorescein diacetate, 10 µM and 1% ethanol, Life Technologies, Stockholm, Sweden, cf.^38^). The loading was thus performed during the last 30 min of the reoxygenation period 30 min before measurements in all conditions. DCF was used since it has previously been applied extensively, including studies of cardiomyocytes^39^. It has some limitation due to interaction with other ROS compounds, but the sensitivity to those is much lower than for H_2_O_2_^40^. DCF thus have limitations, as pointed out by Kalyanaraman et al.^41^, a way to improve the reliability in the measurements would be to include another ROS-probe e.g. the dihydroethidium (HE), but this is also associated with limitations. Dihydroethidium is fully specific for some ROS species^40^ but can require additional techniques (e.g. HPLC) to confirm the oxidation products^41^. After loading the cells were transferred to a cuvette on the stage of an inverted confocal microscope (Zeiss LSM 510 META, Carl Zeiss Microscopy GmbH, Jena, Germany) and perfused with perfusion buffer with 1 mM [Ca^2+^] at room temperature (22 °C). The cells were stimulated via two platinum wires in the cuvette at 0.5 Hz (0.5 ms pulse duration, supramaximal voltage using a Grass S44 stimulator and a current amplifier) and super fused with perfusion buffer (1.0 mM Ca^2+^). The fluorescence intensity (excitation 488, emission 522 nm) was monitored at a frame rate of ~ 0.5 Hz for ~ 10 min. The intensity changes in individual cells were evaluated. The indicator compound is converted to a fluorescent derivative by the action of free reactive oxygen species and we observed a linear increase in intensity in the cells, reflecting production of free reactive oxygen species. The slope of this increase was evaluated by linear regression as a measure of free reactive oxygen species production per unit time. To determine the amount of loaded probe in each cell, 10 µM H_2_O_2_ was added and the maximal fluorescence intensity recorded. This value was used to normalize the fluorescence increase rates. From each batch of cells (i.e. one animal) the recording procedure was performed twice in each condition (normoxia, hypoxia and hypoxia + GLX481304).

### Measurement of intracellular [Ca^2+^] and shortening in cardiomyocytes

The cells were loaded for 30 min in perfusion buffer (1.0 mM Ca^2+^) at room temperature with the Ca^2+^ indicator FLUO-4/AM (6 µM, Life Technologies, Stockholm, Sweden), transferred to the cuvette on the confocal microscope, perfused and stimulated as described above. FLUO-4 was excited at 488 nm and the fluorescence emission recorded with a fully open pin hole using a 522 nm filter. The microscope was run in line-scan mode (frequency 25 Hz) placing the line along the long axis of the cardiomyocyte (cf. Figure 3). Approximate 30 s were recorded for each cell. The maximal fluorescence increase during stimulation (F_max_) was recorded and normalized to the relaxed value immediately before the stimulus (F_0_). The F_max_/ F_0_ ratio was considered to reflect the contraction-associated increase in intracellular [Ca^2+^]. The maximal extent of cell shortening during contraction was determined from the line scans and expressed in percentage of the relaxed muscle length. From each cell, 8 measurements of F_max_/F_0_ and 3 measurement of cell shortening were recorded and the average was taken as representative of the cell. In each experiment 11 to 21 cells were analyzed and the average was taken as representative of the experiment.

### Measurement of mRNA expression with PCR

The polymerase chain reaction (PCR) was used to examine the expression of mRNA for Nox2 (forward primer CCTTTTACCTATGTGCCGGAC; reverse CATGTGATGTGTAGAGTCTTGCT) and Nox4 (forward: TGCCTGCTCATTTGGCTGT; reverse: CCGGCACATAGGTAAAAGGATG). The primers, designed for the mouse Nox forms (5–3’ direction), were obtained using the primer database Primerbank^42^. The products (single bands for each form) were separated on 2% agarose gels.

### Measurements of contractile function of intact hearts

For in vitro measurements of cardiac contraction, a modified Langendorff setup was used^43,44^, with retrograde perfusion via the aorta using 37 °C Krebs–Ringer solution pre-gassed with 95%CO_2_/5% O_2_ (pH 7.4), using a peristaltic pump. The solution was recirculated, passing through filters (mesh: 5 µm after heart and 0.45 µm before the heart), with a total fluid volume of 300 ml in order to minimize the use of Nox inhibitor compound. The heart was held via the perfusion cannula in a warm chamber with humid environment. The perfusion pressure was continuously monitored and maintained at about 80 mmHg (range 80–90 mm Hg, resulting in an initial aortic/coronary flow of about 2 ml/min), by adjusting the pump speed. After the perfusion had been initiated, the left atrium was cut away and a polyethylene balloon was inserted into the left ventricle. The pressure in the ventricle was measured via the water-filled balloon connected to a pressure transducer and a data acquisition system (ADInstruments, Oxford, UK). In a preliminary series of experiments, we determined the optimal filling volume for systolic pressure. In subsequent experiments, we inflated the balloon to this volume (40 µl). All hearts were given a stabilization period of 30 min followed by an ischemia (zero perfusion) period of 30 min. During this time, perfusion was stopped and the surface of the heart was kept moist by a slow superfusion of Krebs–Ringer solution. After the ischemia period, perfusion was restarted and the cardiac function was monitored for 120 min. The hearts were paced during the experiments, except during the ischemia period, at 6 Hz (0.6 ms pulse duration, supramaximal voltage) via electrodes placed on the surface of the heart.

In initial experiments we found that a concentration of 1.25 µM of the Nox inhibitor GLX481304 could be applied without negative effects on contractile function or perfusion pressure. Higher concentrations (2.5–10 µM) resulted in a gradual decay of systolic pressure and increase of diastolic pressure. We do not know the mechanism behind these inhibitory effects at higher GLX481304 concentrations. They may relate to more extensive inhibition of Nox2 and Nox4 activity affecting cell metabolism and/or vasculature properties^6,45^, but we did not examine these effects further. To address the question whether the inhibitor affected the cardiac contractibility and ischemia–reperfusion responses, the hearts were thus exposed, throughout the duration of the experiment, either with Krebs–Ringer solution containing solvent (dimethyl sulfoxide, DMSO 0.1 mM) or the Nox inhibitor (1.25 µM GLX481304, dissolved in DMSO).

### Solutions

*Krebs–Ringer solution (mM)*: 123 NaCl, 4.7 KCl, 1.2 MgCl_2_, 1.2 KH_2_PO_4_, 20 NaHCO_3_, 5.5 D-Glucose, 2.5 CaCl_2_, with pH 7.4 when oxygenated with O_2_/CO_2_ (95%/5%).

*Perfusion buffer (mM)*: 113 NaCl, 4.7 KCl, 0.6 Na_2_HPO_4_, 1.2 MgSO_4_, 12 NaHCO_3_, 10, KHCO_3_, 10 Hepes, 30 Taurine, 5.5 Glucose, with pH 7.46, oxygenated with O_2_/CO_2_ (95%/5%). 2,3-Butanedione Monoxime (BDM) 10 mM was added as indicated in the text. *Digestion enzymes*: 0.125 mg/ml Liberase DH (Roche, Mannheim, Germany), and 0.14 mg/ml Trypsin (Gibco, Life Technologies, Stockholm, Sweden).

### Identification and characterization of NOX inhibitors

GLX481304 was identified (Glucox Biotech, Stockholm, Sweden) using a high-throughput screen, selecting for inhibition of Nox4 activity utilizing a 40 000 chemically diverse library. A 348-well format assay with T-Rex-293 whole cells with inducible Nox 4 overexpression^46^ using Amplex Red-based assay as read-out (fluorescence) identified 728 primary hits with 50% inhibition as threshold. A counter-screen with 4 µM H_2_O_2_ and a re-test using the same assay reduced the number of hits, leaving 93 structurally diverse hits for dose–response investigation. Dose response assay selected the hits from 200 µM, 11 step threefold dilution in duplicate. This resulted in 54 hits that received an IC_50_ and the most potent hits around 1 µM. None of the hits demonstrated any effect of cell viability at 10 µM (Celltitre-Blue assay, resazurin, 24 h; LDH leakage assay, formazan, 3 h). GLX481304 were further characterized regarding iso-form selectivity (Nox1, Nox2 and Nox4) using whole cell assays. Nox1 in CHO cells^47^, Nox2 in human neutrophils^48,49^, Nox4 HEK 293T-Rex^46^ and CJ HEK 293 cells overexpressing Nox4 (purchased from Redoxis, Lund, Sweden). The latter cell type was utilized in a previous publication to determine isoform selective Nox4 activity^50^.

The general methods used for CJ HEK 293, HEK 293 TRex and CHO cells, were to detach the adherent cells by trypsination followed by centrifugation and washing with Hank’s balanced salt solution (HBSS) solution. Cells were then seeded in 96-well black flat-bottom plates at a density of 50,000–100,000 cells/well. All compounds were dissolved in DMSO and concentrations ranging from 0.003 to 200 μM were tested in the Nox cellular assays with a final concentration of DMSO of 1%. Cells were incubated at 37 °C with the compounds for 30 min before measurement. In the high-throughput screen HEK 293 TRex was used and tetracycline (1 mg/ml) was added 18 h before measurement to induce Nox4 expression. Production of hydrogen peroxide by Nox in intact cells was measured using Amplex red fluorescence as described^47^. To examine if GLX481304 inhibited Nox 1, an Amplex red based assay was used on CHO cells overexpressing Nox1 (a kind gift from Dr Vincent Jaquet, Dept. of Pathology and Immunology, Centre Médical Universitaire, Geneva, Switzerland^47^). The ability of GLX481304 to inhibit the iso-form Nox2 was examined using human neutrophils activated with Phorbol 12-myristate (PMA) which stimulates Nox 2 and ROS production, via a protein kinase C and nuclear factor-kappa B pathway. ROS production was determined using isoluminol, a hydrophobic luminescent compound, activated by extra cellular ROS, a reaction amplified by horseradish peroxidase^7^ in the assay. Luminescence after activation with 30 ng/ml PMA was detected using a Fluostar Optima microplate reader (BMG Labtech) with 1 µM Diphenyleneiodonium Chloride (DPI) as a negative control. This assay using isolated neutrophils from whole blood was performed as previously described^48^. To exclude any undesired redox activity GLX481304 was also tested for endogenous redox activity using 2,2-diphenyl-1-picrylhydrazyl-hydrate (DPPH, Sigma Aldrich) assay^51^. Solubility of the Nox inhibitors in DMSO and physiological saline (PBS) was determined using liquid chromatography and mass spectroscopy. Membrane permeability was examined using Caco-2 cell monolayers according to published protocols^52^. The Caco-2 cell monolayers were grown on permeable filter support and used for transport study on day 21 after seeding. Prior to the experiment a drug solution of 10 μM was prepared and warmed to 37 °C. The Caco-2 filters were washed with pre-warmed HBSS prior to the experiment, and thereafter the experiment was started by applying the donor solution on the apical side. The transport experiments were carried out at pH 6.5 in the apical chamber, reflecting the pH of the intestinal lumen, and pH 7.4 in the basolateral chamber reflecting the pH of the blood.

The experiments were performed at 37 °C and with a stirring rate of 500 rpm. The receiver compartment was sampled at 15, 30 and 60 min, and at 60 min also a final sample from the donor chamber was taken in order to calculate the mass balance of the compound. Directly after the termination of the experiment the filter inserts were washed with pre-warmed HBSS and the membrane integrity was checked. This was performed by trans-epithelial electrical resistance (TEER) measurement and by measurement of Mannitol permeability, which is a para-cellular marker used for integrity measurements.

The Caco2 study was performed by a CRO (UDOPP, BMC, and Uppsala) that have the expertise in the field of pharmacological characterization of potential pharmaceutical substances. From this study it was concluded that GLX481304 possess high membrane permeability.

### Statistics

All values are given as Standard Error of the Mean (SEM) with number of observations. Statistical analysis was performed using routines implemented in SigmaPlot 14 for Windows.

### Ethical statement

The study is reported in accordance with ARRIVE guidelines of the European Union Council and the current laws in Sweden.

### Ethics approval

All animal experiments were approved by the Regional Animal Ethical Committee (Stockholm, Sweden) and adhered to the European Community Council Directives (86/609/EEC) and approved by Stockholm’s Norra Djurförsöksetiska Nämnd.

## Results

### Generation and characterization of the Nox inhibitor GLX481304

**Figure 1:**
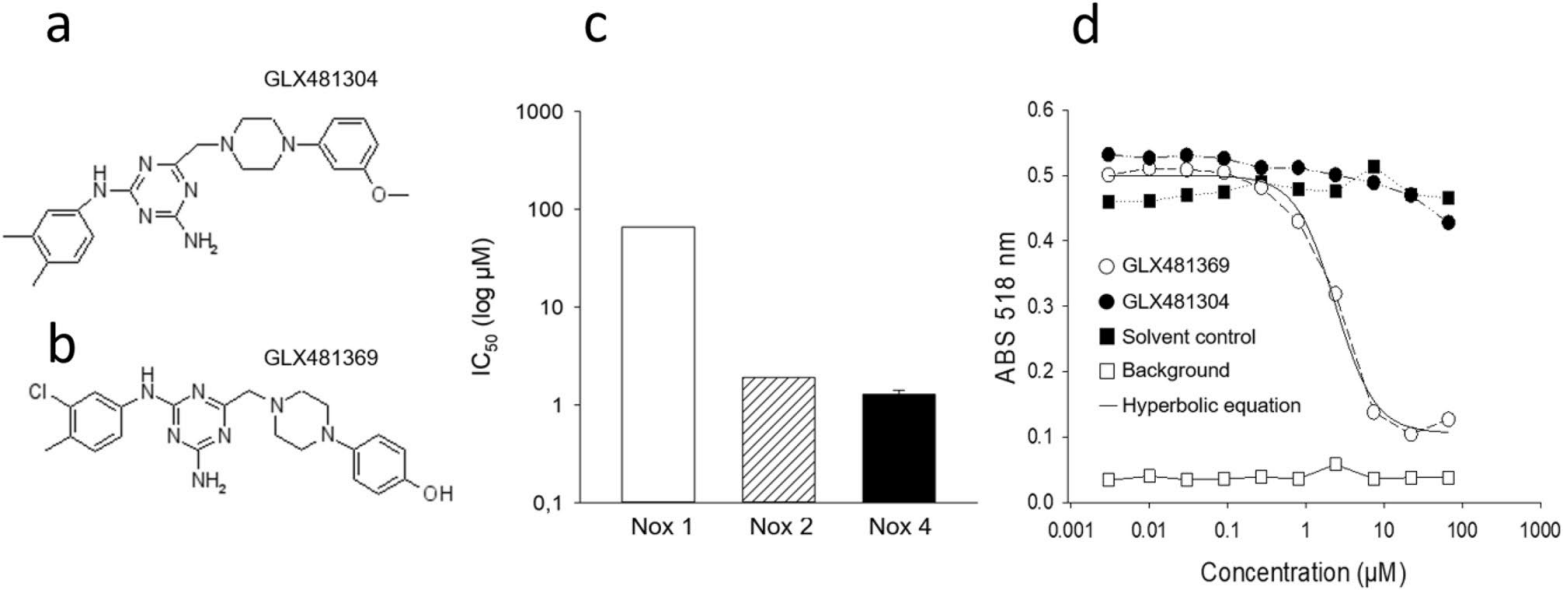
Structure and inhibitory effects of GLX481304 Panel a and b show the chemical structure of GLX481304 and GLX481369 used as a redox control. Panel c show the IC_50_ values of GLX481304 for Nox 1 (open bar, in CHO cells, one technical duplicate), Nox 2 (hatched bar in human neutrophils, one technical duplicate) and Nox 4 (full bar in CJ HEK 293 cells, four measurements) isoforms. The maximal inhibition of Nox4 was 116% and the Hill coefficient 0.94. Panel d shows the antioxidant effects (Y axis shows DPPH absorbance) of the structurally related compound GLX481369 (open circles, with half maximal effect at about 2.3 µM, thin line shows fit to hyperbolic equation) and lack of effects of the GLX481304 (filled circles). The GLX481369 redox active substance was identified and disqualified as a potential Nox4 inhibitor during a SAR development to improve Nox4 inhibitors. During this development GLX481369 was later instead used as control of being a redox active substance in the selection and identification of true Nox4 inhibitors. This substance was previously also used as an internal redox control in^50^. Filled squares shows absorbance in DPPH without added compound and open squares the background fluorescence.

GLX481304 (Panel a, Fig. 1) was identified in a high-throughput screen using inducible overexpressed Nox4 in T-Rex-293 cells and later confirmed with another cell line (HEK 293) that constitutively overexpressed Nox4. Further examination of isoform specificity showed that GLX481304 inhibited both Nox 2 and 4 with low IC_50_ values, whereas it had negligible effects on Nox1 (Panel c, Fig. 1). It was also confirmed, using comparisons with a structurally closely related compound (the redox active substance GLX481369, Panel b Fig. 1) that GLX481304 did not have general antioxidant effects (Panel d, Fig. 1). We have thus shown that GLX481304 is a potent inhibitor of Nox2 and 4 without general antioxidant effects or inhibitory effects on Nox1.

The compound GLX481304 is rapidly taken up over cell membranes in vitro and has a high protein binding ability. Cell permeability was examined using established protocols at UDOPP laboratory, (BMC Uppsala). Permeability in Caco-2 cells, using PAPP A-B assay was 7.4 ± 0.6, 10^–6^ cm/s. This is considered to be a high permeability. It shows no cytotoxicity in HEK 293 cells (Celltitre-Blue assay, 10 µM, 24 h and LDH Leakage assay, 10 µM, 3 h). The Cytoxity Detection Kit Plus (LDH) demonstrated 2.4% cytotoxicity during 24 h and Celltitre Blue Cell Viability assay 75% remaining viable cells for GLX481304 and 6% of the control Chloropromazine. Thus, GLX481304 demonstrated low or no cell toxicity. GLX481304 has low solubility in physiological saline (PBS, about 6 µM), but is easily dissolved in DMSO, our stock solution was 10 mM, the final concentration of DMSO in our experiments was < 0.1%.

### GLX481304 inhibits generation of reactive oxygen species in isolated cardiomyocytes

**Figure 2:**
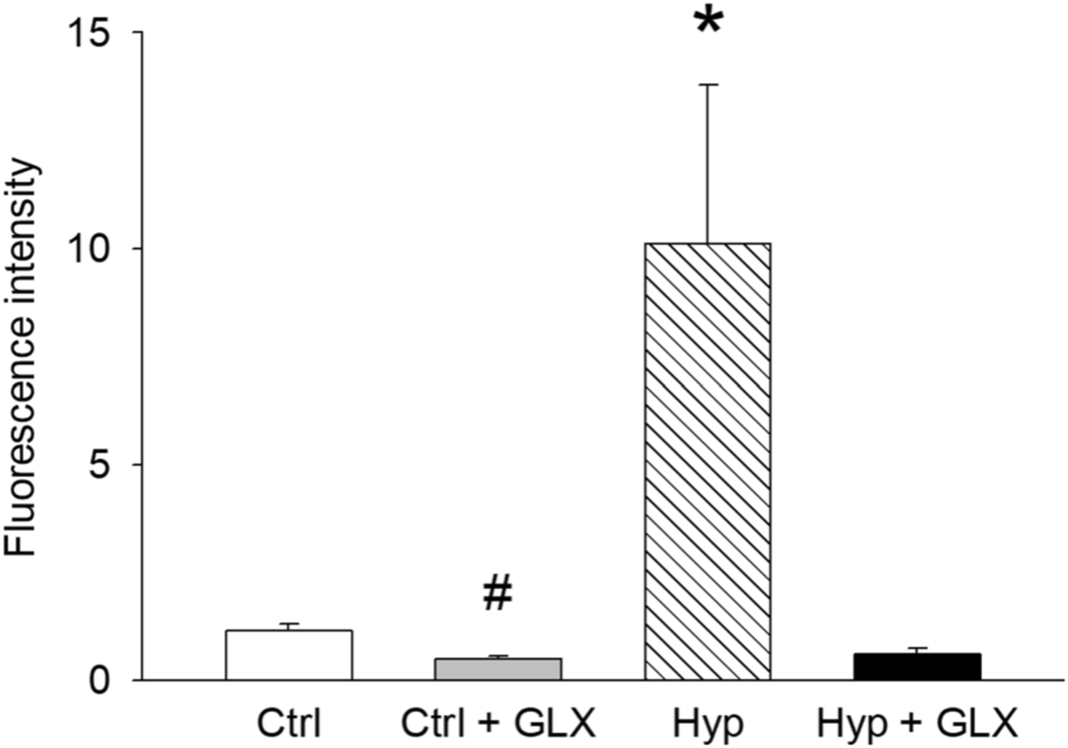
Free Reactive Oxygen Species (ROS) production measured by rate of increase in Carboxy -H_2_DCFDA fluorescence in control cardiomyocytes (Ctrl, open bar, number of cells = 50) and cardiomyocytes exposed to 60 min hypoxia and 2 h reoxygenation without (Hyp. hatched bar, n = 16) and hypoxia reoxygenation in the presence of the GLX481304 (Hyp + GLX481304, filled bar, n = 23). Gray bar shows effects of GLX481304 on cell batches without hypoxia induced ROS production (n = 18). The cells were obtained from three separate batches of cell isolation, i.e. animals. Each batch was exposed to the different treatment conditions. * indicates statistical difference (P < 0.05) compared all other groups and # P < 0.05 compared to the controls. (ANOVA on log values with Holm-Sidak method for multiple comparisons).

We initially confirmed, using PCR (on 5 independent samples), that mRNA of both Nox 2 and Nox 4 are expressed in the mouse heart tissue, consistent with previous data^26^. We then explored if inhibition of Nox 2 and Nox 4 with GLX481304 affected the Reactive Oxygen Species (ROS) generation in isolated cardiomyocytes from different cell batches (i.e. animals). The cells were exposed to either normoxia (controls) or hypoxia in low glucose (mimicking ischemia) for 60 min followed by 2 h reoxygenation and exposure to glucose, and subsequently loaded with the fluorescent reactive oxygen species indicator Carboxy-H_2_DCFDA. A clearly increased measurable ROS production (reflecting a hypoxia/reoxygenation challenge and sufficient loading of the indicator) was observed in 3 of 6 cell batches and the effects of GLX481304 was examined in these experimental groups (including >  = 16 cells). Figure 2 shows mean values of the fluorescence increase from the indicator probe in the different conditions. Cell batches responding to the hypoxia-reoxygenation treatment with an increase in production of reactive oxygen species (hatched bar, Fig. 2), had clearly inhibited ROS production in the presence of GLX481304 (black filled bar in Fig. 2). In cells loaded with Carboxy -H_2_DCFDA, but not expressing high ROS, GLX481304 inhibited the ROS production slightly (grey bar, Fig. 2) compared to the non-hypoxic controls (open bar, Fig. 2). These results thus show that both Nox2 and 4 are expressed, and that inhibition of these two enzymes leads to attenuated ROS levels after a hypoxia challenge in the mouse cardiomyocytes.

### GLX481304 improves cardiomyocyte contractility after hypoxia-reoxygenation

**Figure 3:**
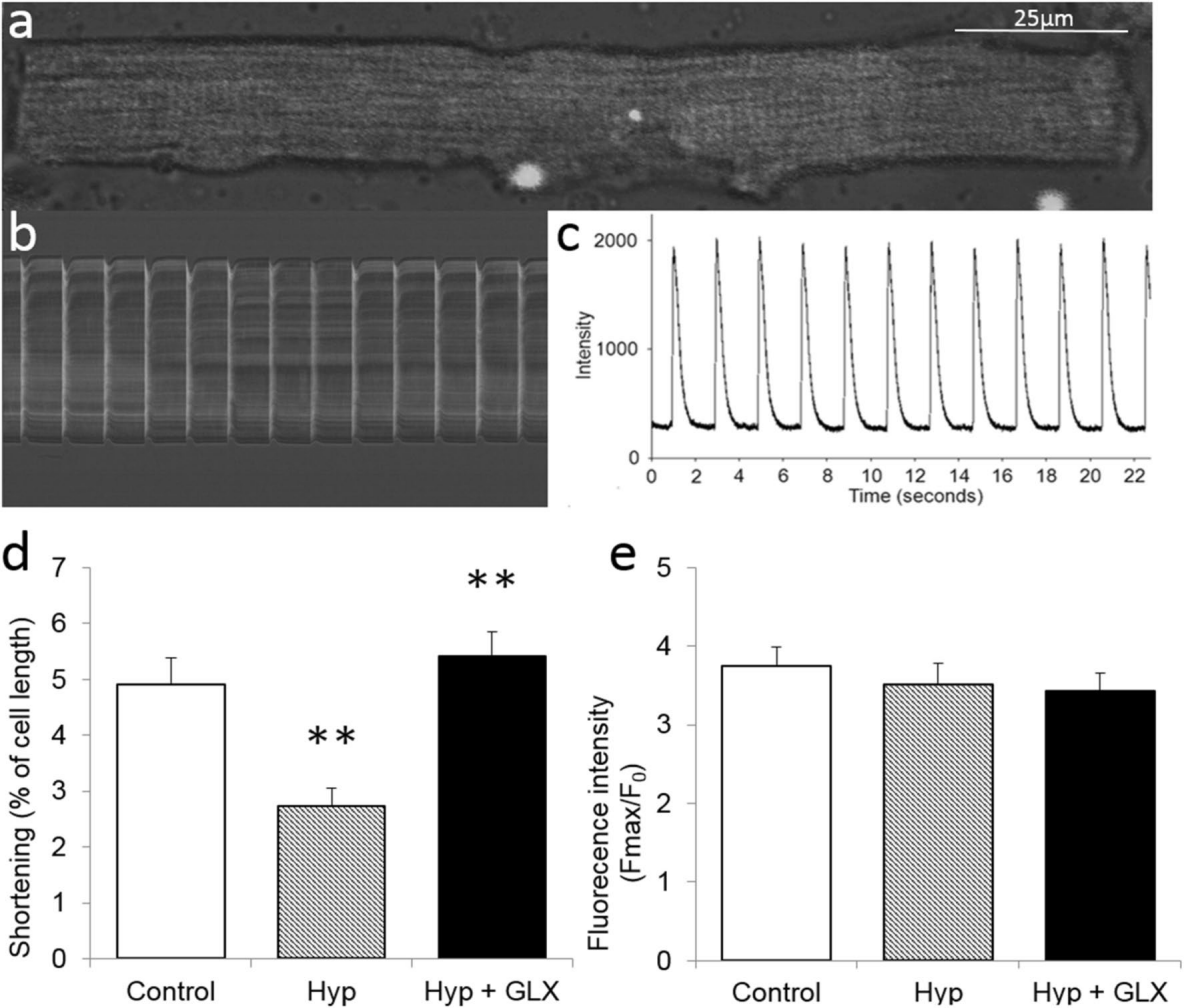
Panel a shows a confocal line scan through a control cardiomyocyte loaded with Fluo-4. Panel b shows the intensity vs time and Panel c the integrated intensity of the fluorescence signal, reflecting changes in intracellular [Ca^2+^]. Panel d shows shortening responses and Panel e the contraction associated increase in fluorescence of stimulated isolated cardiomyocytes exposed to normoxia (control open bar, n = 12) or hypoxia/reoxygenation without (Hyp, grey bar, n = 11–13) or with (Hyp + GLX481304, 1.25 µM, filled bar, n = 20–21). ** P < 0.01 vs both controls and Hyp + GLX481304 group (ANOVA and Holm-Sidak method).

To address the question whether the effects of GLX481304 on reactive oxygen species production in the cardiomyocytes exposed to hypoxia-reoxygenation was accompanied by improved contractile performance we examined shortening responses and Ca^2+^ transients in isolated cardiomyocytes exposed to the hypoxia-reoxygenation challenge. Panel a of Fig. 3 shows an isolated control mouse cardiomyocyte with the confocal scan line used for measurements indicated. In panel b the resulting series of scan lines in a stimulated cell are shown and panel c depicts the integrated intensity changes during a series of stimulations. The hypoxia-reoxygenation challenge gave a clear reduction in cell shortening responses (Panel d). The GLX481304 Nox inhibitor significantly improved the cardiomyocyte shortening responses after the hypoxia-reoxygenation challenge. No significant change in intracellular Ca^2+^ levels could be seen between the groups. These data suggest that the contractile impairment after hypoxia-reoxygenation under these conditions and the beneficial effects of GLX481304 are not mediated by alterations in the Ca^2+^ translocation.

### GLX481304 improves whole heart contractility after hypoxia-reoxygenation

**Figure 4:**
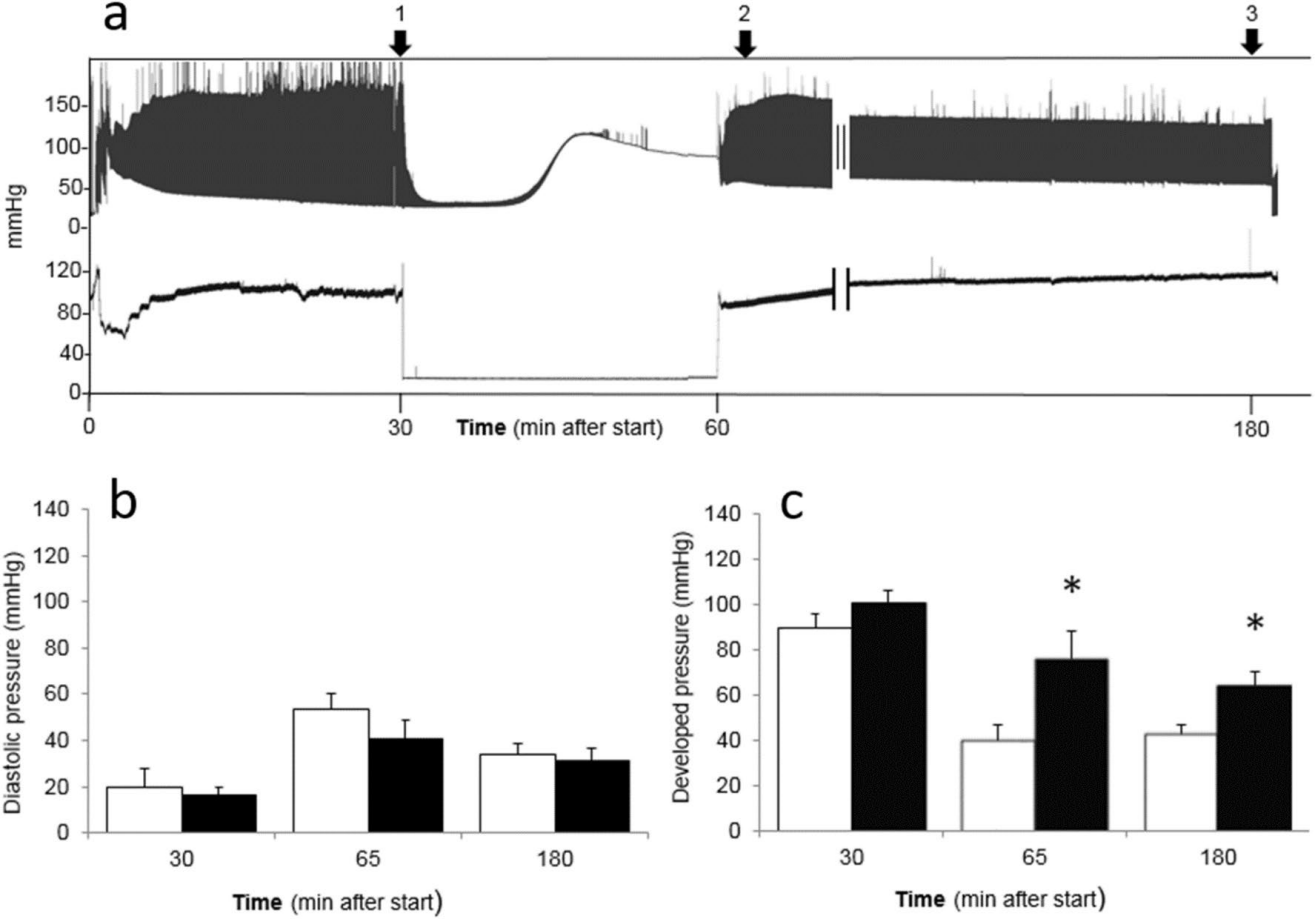
Effects of GLX481304 on pressure responses in Langendorff perfused mouse hearts. Panel a shows the ventricular pressure changes and panel b the perfusion pressure in the aorta of a control heart. Data were obtained at 30 min after start (arrow 1), immediately after start of reperfusion at 65 min (arrow 2) and after two hour’s reperfusion at 180 min (arrow 3). The hypoxia (non-perfused) period is 30 min (between arrows 1 and immediately before 2), followed by reperfusion. Panel b shows the diastolic pressure and panel c the developed pressure, before (arrow 1), immediately (5 min, arrow 2) and late (2 h, arrow 3) after onset of reperfusion in the non-treated control group (open bars) and in hearts receiving GLX481304 (1.25 µM, filled bars), n = 7–8. * P < 0.05 (Two Way Repeated Measures ANOVA with Holm-Sidak method for comparison).

We evaluated the effects of the GLX481304 in the intact isolated beating heart using a Langendorff setup. Figure 4 shows an original recording of cardiac ventricular pressure (upper trace in Panel a) and perfusion pressure (lower trace in Panel a) during an in vitro ischemia–reperfusion experiment in an isolated perfused mouse heart. Immediately after mounting of the preparation the systolic (active) pressure increased and the diastolic (relaxed) pressure decreased. The values stabilized within about 15 min and initial values, prior to the ischemia (i.e. un-perfused) period, were recorded at 30 min (first arrow in Panel a of Fig. 4). When perfusion was stopped, to mimic ischemia, the active contractions ceased within a minute and after about 10 min a sustained pressure developed. When the perfusion was restarted the sustained contraction disappeared and within about 5 min, a lower diastolic pressure was established with superimposed active contractions. The diastolic and systolic pressures were recorded during two hours after the ischemia period and pressure values were determined 5 and 120 min after onset of reperfusion (second and third arrows). At the start of the experiment the perfusion pressure was adjusted to at about 90 mmHg resulting in about 2–3 ml/min perfusion. At 5 min after start of the reperfusion (2nd arrow in Panel a of Fig. 4) the perfusion pressure tended to be lower and the perfusion flow higher in the GLX481304 treated group (controls: 88.6 ± 5.1 mmHg; 2.3 ± 0.3 ml/min, n = 8; GLX481304 treated 80.9 ± 1.8 mmHg; 3.1 ± 0.24 ml/min, n = 7). The vascular resistance (calculated as pressure/flow; controls: 43.9 ± 6.6 mmHg min/ml, n = 8; GLX481304 treated 27.5 ± 2.6 mmHg min/ml, n = 7) was significantly (P < 0.05) lower in the GLX481304 group at 5 min after onset of reperfusion. At the end of the experiment, 120 min after start of reperfusion (third arrow in Panel a of Fig. 4) the perfusion pressure was slightly lower and flow slightly higher in the GLX481304 group (controls: 95.4 ± 2.7 mmHg; 1.6 ± 0.3 ml/min. n = 8; GLX481304 treated 93.6 ± 1.3 mmHg; 2.2 ± 0.3 ml/min, n = 7). The resistance values remained lower in the GLX481304 group at 120 min (controls: 78.2 ± 15.2 mmHg min/ml, n = 8; GLX481304 treated 47.8 ± 6.6 mmHg min/ml, n = 7). Although the effects of GLX481304 at 5 min after reperfusion remained to some extent for 120 min, the differences in pressure, flow and resistance were however not significant between controls and the GLX481304 this at the later time point. The results thus show that the GLX481304 treatment gave a significantly lower flow resistance with largest effects initially after the ischemia/reperfusion.

Panel’s b and c of Fig. 4 summarizes the results from the ischemia–reperfusion experiments in the intact hearts. The diastolic pressure (Panel b) was increased after 5 min of reperfusion following the ischemia period in both the GLX481304 and the control groups. The mean values tended to be lower in the GLX481304-treated group, although no significant difference could be detected. The active developed pressure (i.e. systolic minus diastolic, Panel c) after ischemia–reperfusion was markedly decreased in the non-treated controls. In the non-treated control group the developed pressure after 5 min reperfusion was about 46% (40.1 ± 6.8 mmHg, n = 8) of the initial before the ischemia–reperfusion period. In contrast, the GLX481304-treated group was significantly (P < 0.05) less affected and the corresponding developed pressure value was about 75% (76.0 ± 12.1 mmHg, n = 8) of the initial. The developed pressure values at both 5 min and 120 min of reperfusion were significantly higher in the GLX481304 group compared to the controls. These results thus show that the presence of the GLX481304 Nox inhibitor significantly improved the contractile function and resistance to flow after an ischemia–reperfusion challenge in the isolated mouse heart.

## Discussion

We have identified a novel compound (GLX481304) with selective inhibitory effects on Nox 2 and 4, both expressed in mouse cardiac tissue. GLX481304, promptly reduces the free reactive oxygen species generation in cardiomyocytes and improves contractile function in cells and whole heart after a hypoxic/”ischemic “- reperfusion challenge.

Generation of Reactive Oxygen Species (ROS) via Nox enzymes is an important event in ischemia–reperfusion injury in the heart^3^, but the detailed pathophysiological impact of Nox activity is not fully understood, and in some aspects controversial: Significantly decreased injury following myocardial ischemia/reperfusion has been reported in Nox 1 and Nox 2 knockout mice, but not in Nox 4 deficient animals^33^. It has been shown^34^ that Nox 4 knockout decreases activation of autophagy in cardiomyocytes, an essential survival mechanism after energy stress, following glucose deprivation. Furthermore, endothelial over-expression of Nox 4 enhances endothelial vasodilation^53^. On the other hand^6^, reported that systemic knockdown of Nox 2 or systemic and cardiac knockdown of Nox 4 significantly reduced infarct size and area at risk after ischemic reperfusion challenge. Interestingly, a double knockout of Nox 2 and 4 and overexpression of a dominant negative Nox (reducing both Nox isoforms) increased the injury^6^. A study by^54^ showed that both overexpression of Nox 4 and reduced Nox activity by expression of a dominant negative forms impaired contractile function after ischemia reperfusion. Down regulation of Nox 4 using a plant derived flavonoid has also been shown to reduce chemical injury to isolated cardiomyocytes^55^. The available data thus suggest a complex interplay between the Nox isoforms, and that both protective and destructive processes can be initiated depending on activation state of the different enzymes, processes difficult to mimic using knockdown strategies. It thus seems logical to conclude that results from knockdown animal models of either of these enzymes are difficult to interpret and not possible to directly translate to in vivo/clinical conditions. An isoform selective and graded pharmacological inhibition of the Nox enzymes, would therefore be a more relevant approach to understand their functions and to possibly combat ischemia–reperfusion injury in heart and other tissues.

It is clearly quite a challenge to develop specific and iso-form selective NOX inhibitors. Lately four publications have been investigating the quality of several of the commonly used NOX inhibitors available today^56–58^. Several of these inhibitors investigated were found to have redox and ROS scavenging activities and/or not being iso-form selective.

Currently, few compounds selective for the different Nox isoforms are available and characterized. General inhibition can be obtained using, e.g. apocynin and diphenyleneiodonium (DPI), but these compounds are not selective for Nox and have effects on several other enzyme systems^59^. ML171 is presented as a NOX1 inhibitor with a 10–20 times selectivity in relation to NOX2, NOX3, NOX4, Glucose oxidase and Xanthine oxidase^60,61^. The mechanism of action was being questioned in one study for ML171 NOX1 inhibition^62^. VAS 3947 and VAS2870 have been introduced as more specific inhibitors of all Nox isoforms, but also these compounds can have off-target effects^59^. In spite of this imperfection VAS2870 is proposed to be a pan inhibitor^63^ of NOX isoforms. VAS2870 has been demonstrated to have several beneficial effects in preclinical disease models, including stroke, pulmonary hypertension, thrombosis, Alzheimer’s disease^56^. Compounds with isoform specificity have also been developed, e.g. the GKT136901 and GKT137831 which inhibits Nox 1 and 4. GTK137831 has been shown to rescue heart function after ischemia/reperfusion in mice^64^. Recently the mechanism of action regarding NOX inhibition by GKT136901 and GKT137831 have been questioned when stringent testing was performed^65^. GKT137831 is in spite this going through phase 2 clinical trials in diabetic nephropathy targeting NOX1/NOX4 activity. Here we show that GLX481304 in whole cell assays, expressing the NOX isoforms NOX1, NOX2 and NOX4, has a clear selectivity and GLX481304 clearly passed the test of not being redox active or ROS-scavenging.

Thus, although the detailed action of Nox inhibitors is not fully clarified, beneficial results using Nox inhibitors, e.g. DPI, Apocynin, VAS 2870^5,66,67^ have been reported in animal models of ischemic stroke, suggesting that pharmacological inhibition of this enzyme system is a promising strategy for treating ischemic conditions in the brain. Beneficial results were also demonstrated when ML171, VAS2870 and the completely iso-form selective GLX7013114^50^ were utilized to clarify the different roles of NOX isoforms of the pathophysiology in the retina caused by AMPA induced excitotoxicity^68^.

To our knowledge, studies of pharmacological Nox inhibition in ischemia/reperfusion challenge to the heart are very limited. As pointed out by Cadenas^3^, ROS signalling can be both protective and harmful for the heart and a carefully graded pharmacological inhibition of Nox would therefore be an attractive clinical approach to target the negative effects of ischemia/reperfusion. The specific Nox 4 inhibitor GLX7013114 has demonstrated protective effects on pancreatic islet cells and on retinal cells^50,68^.

The brain seems to be particularly sensitive to Nox 4 induced cytotoxicity^69^, but as discussed above the cardiac system might be potentially affected by both Nox 4 and 2. We therefore decided, in the present study, to target these two main Nox isoforms (2 and 4) proposed to be involved in ischemia/reperfusion injury of the heart^6^. Our characterization of the GLX481304 demonstrated selectivity for these two Nox isoforms and activity with IC_50_ values in the micromolar range.

ROS/Nox have several targets in the vascular smooth muscle and vascular endothelium^53,70^. However, GLX481304 clearly inhibited the up-regulated ROS in the post ischemic isolated cardiomyocytes and resulted in a significant attenuation of the ischemia induced contractile impairment, which identifies the cardiac muscle as a key target. The cardiomyocyte contraction was inhibited after the ischemia/reperfusion with lowered shortening despite normal Ca^2+^ transients. This implies that ROS induced inhibition in the cardiomyocytes occurs via steps down-stream of the Ca^2+^ increase, e.g. via effects on the cell energy metabolism, thin filament regulation or the contractile function it self. The effects of GLX481304 on ROS production in the cardiomyocytes was most pronounced after ischemia/reoxygenation whereas effects on non-upregulated ROS was lower (Fig. 2). Effects on contractility (Fig. 4) in normoxia was minimal, whereas it partially rescued cardiomyocytes and heart after hypoxia/reoxygenation (Figs. 3, 4). This suggests that the effects are mediated via effects on increased ROS production. The ROS hydrogen peroxide (H_2_O_2_) is well known as an intermediate in cellular signaling as it will bind to certain amino acids (cysteine, methionine) of proteins^71^. It has a long biological life span and is capable of membrane diffusion between cells^72^. It has been shown that reactive oxygen species reduce contractile force and reduces Ca^2+^ sensitivity in permeabilized pig cardiac muscle fibers, possibly by direct effects on the contractile system^73^. We therefore conclude that the beneficial effects of GLX481304 in the whole heart (Langendorff perfused) system are to a large extent due to direct effects on the cardiomyocytes by preventing a ROS induced modification of the contractile system.

## Conclusions

We have shown that Nox2/Nox4 inhibition is a potentially useful pharmacological treatment of ischemia/reperfusion induced cardiac dysfunction. The inhibition of Nox2/Nox4 in the cardiac tissue using the GLX481304 is most likely only partial, a total knockout would, as previously shown in genetically modified animal, give less predictable results and possibly also reductive stress by the complete lack of Nox activity, which most likely can be as detrimental as oxidative stress. Future pharmacological treatment attempts, e.g. in myocardial ischemia/infarction, or during potential other challenges to the cardiac system including transplantation, must be performed within a therapeutic window. Our results also suggest that it is probably not necessary to have strict selectivity to one Nox isoform in the cardiac system.
